# Restoration of HDAC1 Enzymatic Activity after Stroke Protects Neurons from Ischemia/Reperfusion Damage and Attenuates Behavioral Deficits in Rats

**DOI:** 10.3390/ijms221910654

**Published:** 2021-09-30

**Authors:** Jui-Sheng Chen, Hao-Kuang Wang, Yu-Ting Su, Chien-Yu Hsu, Jia-Shing Chen, Cheng-Loong Liang, Cheng-Chun Wu, Aij-Lie Kwan

**Affiliations:** 1 Graduate Institute of Medicine, College of Medicine, Kaohsiung Medical University, Kaohsiung 807, Taiwan; dumboschen@gmail.com; 2 Department of Neurosurgery, E-Da Dachang Hospital, I-Shou University, Kaohsiung 807, Taiwan; 3 Department of Neurosurgery, E-DA Hospital, I-Shou University, Kaohsiung 824, Taiwan; ed101393@edah.org.tw (H.-K.W.); a0970598731@gmail.com (C.-Y.H.); p0201@edah.org.tw (C.-L.L.); 4 School of Medicine, College of Medicine, I-Shou University, Kaohsiung 824, Taiwan; 5 School of Medicine for International Students, College of Medicine, I-Shou University, Kaohsiung 824, Taiwan; jasonchen1228@isu.edu.tw; 6 Department of Obstetrics and Gynecology, Kaohsiung Chang Gung Memorial Hospital and Chang Gung University College of Medicine, Kaohsiung 833, Taiwan; kimyy9487@cgmh.org.tw; 7 Department of Neurosurgery, Kaohsiung Medical University Hospital, Kaohsiung 833, Taiwan

**Keywords:** HDAC1, stroke, DNA damage, apoptosis, mNSS, cylinder test

## Abstract

A therapeutic approach for promoting neuroprotection and brain functional regeneration after strokes is still lacking. Histone deacetylase 1 (HDAC1), which belongs to the histone deacetylase family, is involved in the transcriptional repression of cell-cycle-modulated genes and DNA damage repair during neurodegeneration. Our previous data showed that the protein level and enzymatic activity of HDAC1 are deregulated in stroke pathogenesis. A novel compound named 5104434 exhibits efficacy to selectively activate HDAC1 enzymatic function in neurodegeneration, but its potential in stroke therapy is still unknown. In this study, we adopted an induced rat model with cerebral ischemia using the vessel dilator endothelin-1 to evaluate the potential of compound 5104434. Our results indicated compound 5104434 selectively restored HDAC1 enzymatic activity after oxygen and glucose deprivation, preserved neurite morphology, and protected neurons from ischemic damage in vitro. In addition, compound 5104434 attenuated the infarct volume, neuronal loss, apoptosis, DNA damage, and DNA breaks in cerebral ischemia rats. It further ameliorated the behavioral outcomes of neuromuscular response, balance, forepaw strength, and functional recovery. Collectively, our data support the efficacy of compound 5104434 in stroke therapy and contend that it can be considered for clinical trial evaluation.

## 1. Introduction

A stroke, also called a cerebrovascular accident, is the rapid loss of brain function due to disturbance in the blood supply to the brain. Currently, it is one of the most lethal diseases. It was the second leading cause of death worldwide in 2016, accounting for 10.1% of deaths [1]. Because of its high morbidity and mortality rates, a stroke causes physical and physiological stress to individuals and imposes high economic burdens on families and social resources.

Ischemic stroke is the major stroke type [2] caused by the mechanical blockage of intracerebral blood vessels, leading to brain tissue damage at the distal region due to a lack of adequate blood and oxygen supply. This mechanical blockage results from in situ atherosclerosis, thrombus (blood clots), or distal embolism caused by cardiac arrhythmia, fat, air, tumor, or even bacterial clusters. At present, essential therapeutic strategies for stroke are limited. The primary therapeutic approach for stroke is to remove the blockage as soon as possible after a stroke episode. To treat acute ischemic stroke, thrombolytic agents with recombinant tissue plasminogen activator have been used intravenously within 3 h of a stroke episode [3,4,5,6]. Another common approach for treating an acute ischemic stroke is endovascular mechanical thrombectomy within 6 h of the stroke episode [7,8,9,10]. Stroke-induced neuronal loss and neurological dysfunctions are common causes of death and disability today. Energy-depletion-mediated accumulation of excitotoxicity reagents and reactive oxygen species as well as inflammation-induced secondary injuries to the ischemic brain are involved in the pathogenesis [11], all of which contribute to neuronal death progression, making treatment challenging. An appropriate therapy to improve the outcome of excessive neuron loss following the irreversible pathogenesis of ischemic damage is difficult to develop [12].

Histone plays a crucial role in posttranslational modification, altering the interaction between DNA and nuclear proteins as transcriptional repression. Histone acetylation levels are dynamically balanced by histone acetyltransferases and histone deacetylases (HDACs), which regulate target gene transcription through chromatin conformation modulation. Generally, HDACs promote chromatin compaction and function as a transcriptional repressor to repress gene expression [13,14]. In the HDAC family, HDAC1 is a member of class l HDACs and is expressed in the brain [15,16]. In neurodegeneration, HDAC1 deregulation is involved in cell cycle aberration and is associated with DNA damage [17]. In the DNA damage repair mechanism, HDAC1 participates in DNA damage response through interaction with FUS [18]. HDAC1 also potentially modulates glial cell reactivation in inflammation [19] and controls neuronal death or survival through interaction with HDAC3 [20]. Remarkably, our recent findings showed that the levels of HDAC1 and its enzymatic activity are decreased in stroke pathogenesis. HDAC1 inhibition exacerbated damages in terms of infarct volume, neuronal loss, DNA damage, neuronal apoptosis, levels of reactive oxygen species, and inflammation cytokines [21].

Nonselective pan-HDAC inhibitors (pan-HDACis) can mitigate neuronal injury and have improved functional outcomes in multiple preclinical models of focal ischemia [22,23]. For example, valproic acid and sodium butyrate treatments target HDAC classes I and IIa [24,25,26,27,28]; suberoylanilide hydroxamic acid treatment targets HDAC classes I, IIa, IIb, and IV [26]; and trichostatin treatment targets HDAC classes I, IIb, and IV [29]. The toxicity of pan-HDACi may affect the diverse central nervous system cell types in unexpected ways [30,31], increasing the risk of unknown toxicity and side effects.

This study adopted an ischemic cerebral stroke model with endothelin-1 induced through stereotaxic injection [32,33] and examined the effect of a specific HDAC1 activator (HA), compound 5104434, delivered through intraperitoneal injection. In our previous study, we identified compound 5104434 as a novel HDAC activator specifically targeting HDAC1 among several HDAC members from the class I family, such as HDAC2, HDAC3, HDAC8 [34]. It possesses neuroprotective potential for neurons with DNA damage in a neurodegenerative mouse model of TDP-43 proteinopathy through its efficacy and specificity for HDAC1 enzymatic activation [34]. Due to the role of HDAC1 in stroke pathogenesis being largely unknown, we examined the interplay of HDAC1 and evaluated the therapeutic potential of compound 5104434 in stroke treatment.

## 2. Results

### 2.1. Increased HDAC1 Enzymatic Activity Preserves Neuronal Survival and Ameliorates Reactive Oxygen Species and LDH Production In Vitro

**Figure 1:**
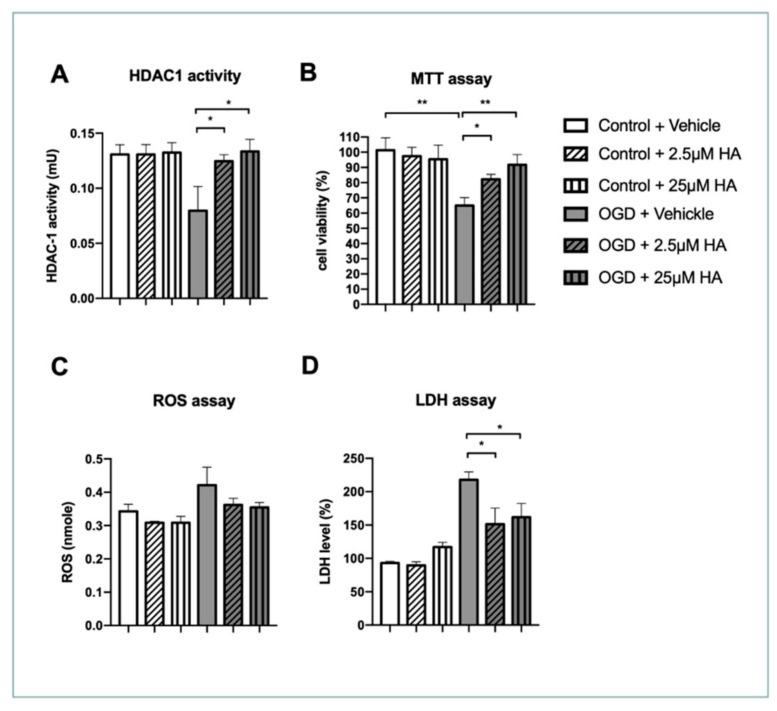
HA treatment restored HDAC1 enzymatic activity and protected neuronal damage after OGD in vitro. (**A**) HDAC1 activity assay was conducted 24 h after OGD. HDAC1 was isolated from protein lysates extracted from primary neuron by immunoprecipitation, and then HDAC1 activity was detected by the assay kit. (**B**) Cell viability was evaluated by MTT assay 24 h after OGD. (**C**) Protein lysates extracted from primary neuron were subjected to ROS assay 24 h after OGD. (**D**) Neuronal damage was evaluated by LDH assay 24 h after OGD. *n* = 6. * Denotes *p* < 0.05, ** denotes *p* < 0.01.

To examine the effect of specifically increased HDAC1 activity in neuroprotection after ischemic insult, we conducted a cortical neuron primary culture and subjected neurons to oxygen and glucose deprivation (OGD). Then, we performed HDAC1 activity, conducted an MTT assay, and estimated reactive oxygen species (ROS) and LDH levels. We evaluated the dose–response of the HDAC1 activator (HA), compound 5104434, at 2.5 and 25 μM. HDAC1 activity assay results showed that OGD considerably affected the HDAC1 enzymatic activity 24 h after ischemic insult, whereas HA treatment supported neurons to maintain HDAC1 enzymatic activity, particularly at a high dose (Figure 1A). Furthermore, a cell viability assay showed that HA promoted neuronal viability following OGD (Figure 1B) and attenuated ROS and LDH levels in cultured cortical neurons (Figure 1C,D). Consequently, our in vitro data indicated that restoring HDAC1 enzymatic function by the compound 5104434 is essential for protecting neurons from OGD insult.

### 2.2. Increased HDAC1 Enzymatic Activity Conserves the Complexity of Neurite Outgrowth in OGD Neurons

**Figure 2:**
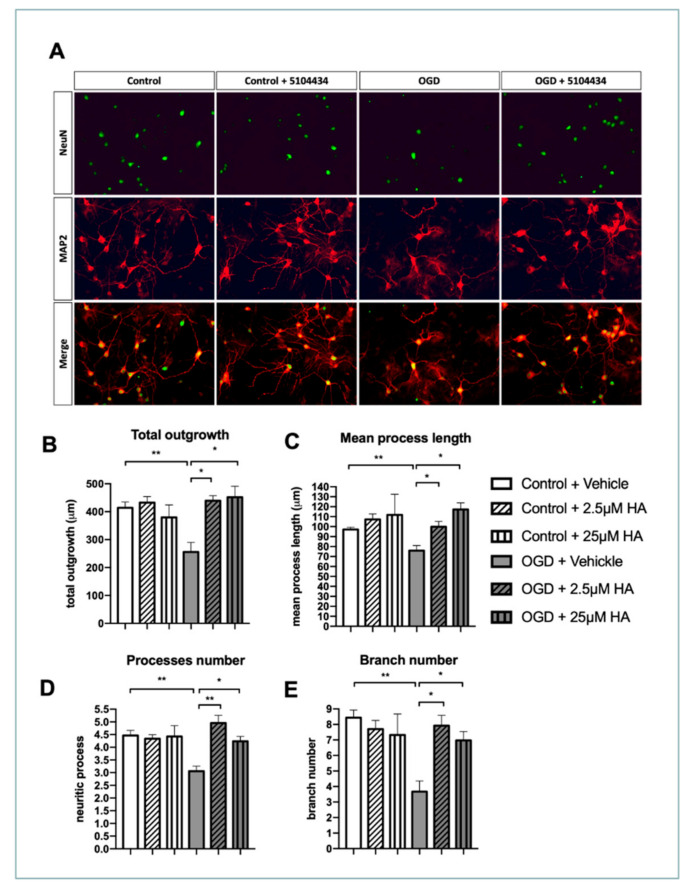
HA treatment preserved neurite outgrowth pattern in terms of total outgrowth, process length, process number, and branch number in vitro. (**A**) Representative figures of immunocytochemistry staining for NeuN and MAP2 in primary neuron 24 h after OGD. (**B**–**E**) High-throughput-acquired neuron images were analyzed in terms of total outgrowth, process length, process number, and branch number. *n* = 6. * Denotes *p* < 0.05, ** denotes *p* < 0.01.

Neuronal function and status are highly associated with neurite complexity, regulated through complex intracellular signaling events [35,36]. Therefore, we evaluated the neurite outgrowth pattern in terms of total neurite length, process number, and branch number in cultured cortical neurons. With immunofluorescent staining for Map2 and NeuN, neuronal morphology could be identified and analyzed based on its neuritis characters. Our study demonstrated that ischemic insult considerably damages the neurite outgrowth pattern, suggesting that OGD is crucial to reduce neuronal survival and neurite outgrowth (Figure 2A). However, HA treatment protected the neuronal number and injured neurite outgrowth pattern. According to quantitative data, HA treatment ameliorated neurite damage in terms of total neurite outgrowth, mean process length, process number, and branch number of culture neurons with OGD (Figure 2B–E). These data suggest that HA is a feasible solution for protecting neurons from OGD insult to preserve neurite complexity.

### 2.3. HA Treatment Restores HDAC1 Activity and Attenuates Brain Damage after Cerebral Ischemia

**Figure 3:**
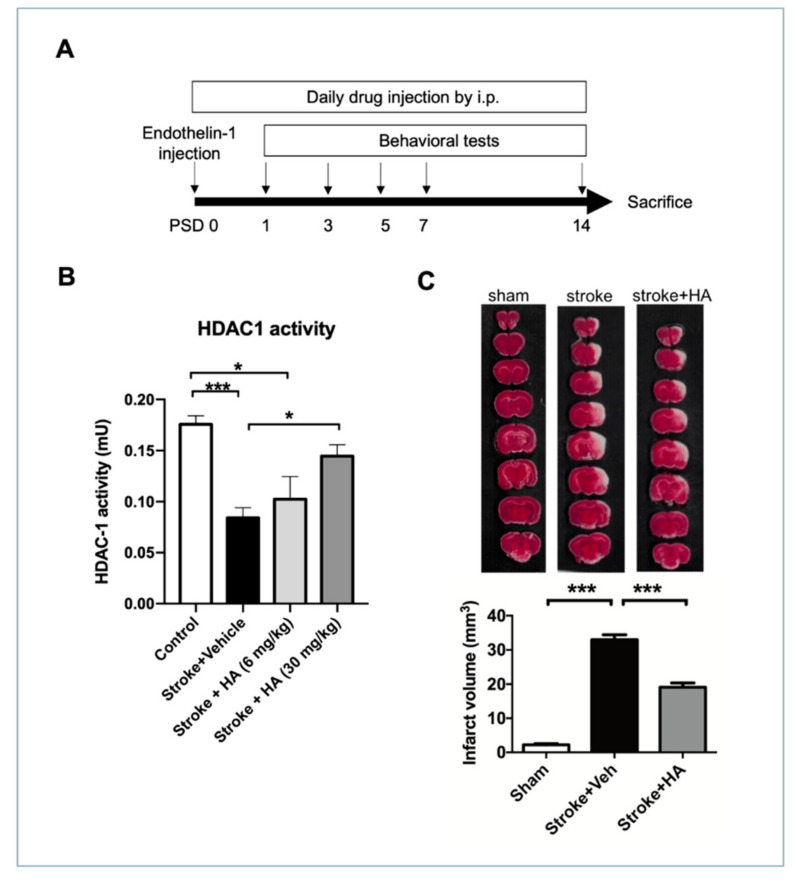
HA treatment attenuated HDAC1 enzymatic dysfunction and ischemia volume of the brain in rats. (**A**) Flow chart of experimental design in vivo. (**B**) HDAC1 enzymatic activity assay for evaluation of the dose–effect of HA. *n* = 6 (**C**) Representative figures of TTC staining that were conducted at post-stroke day 3 in rats with endothelin-1-induced brain ischemia. Quantified data of ischemia volume from rats with brain ischemia. *n* = 8. * Denotes *p* < 0.05; *** denotes *p* < 0.001.

To evaluate the efficacy of HA in protecting the brain from damage after stroke, we used an endothelin-1 induced rat model of cerebral ischemia and HA treated daily with i.p. (compound 5104434, 30 mg/kg/day [34]) since the first day after surgery. To evaluate the efficacy of HA, we conducted series of behavioral tests on post-surgery days (PSDs) 1, 3, 5, 7, and 14 (Figure 3A). Firstly, we evaluated the dose–effect of HA in vivo referenced by our previous study through an HDAC1 enzymatic activity assay. The data showed that the dose of 30 mg/kg could sufficiently activate its activity, but 6 mg/kg could not (Figure 3B). Accordingly, we adopted the amount of 30 mg/kg for the following evaluations in this study. To understand the effect of HA in neuronal viability, we performed triphenyl tetrazolium chloride (TTC) staining to detect brain parenchyma with mitochondrial activity to assess the infarct volume at 3 days after the stroke episode (Figure 3C). The quantitative data indicated that rats with ischemic stroke exhibited significant infarct volume compared with sham rats, whereas HA treatment prevented neuronal loss in the pathogenesis of an ischemic stroke, reducing the total ischemic volume in the rats with cerebral ischemia. Thus, restoration of HDAC1 enzymatic activity was shown to elicit a neuroprotective effect in preventing neuronal loss after a stroke.

### 2.4. HA Decreases Neuronal Apoptosis after a Stroke

**Figure 4:**
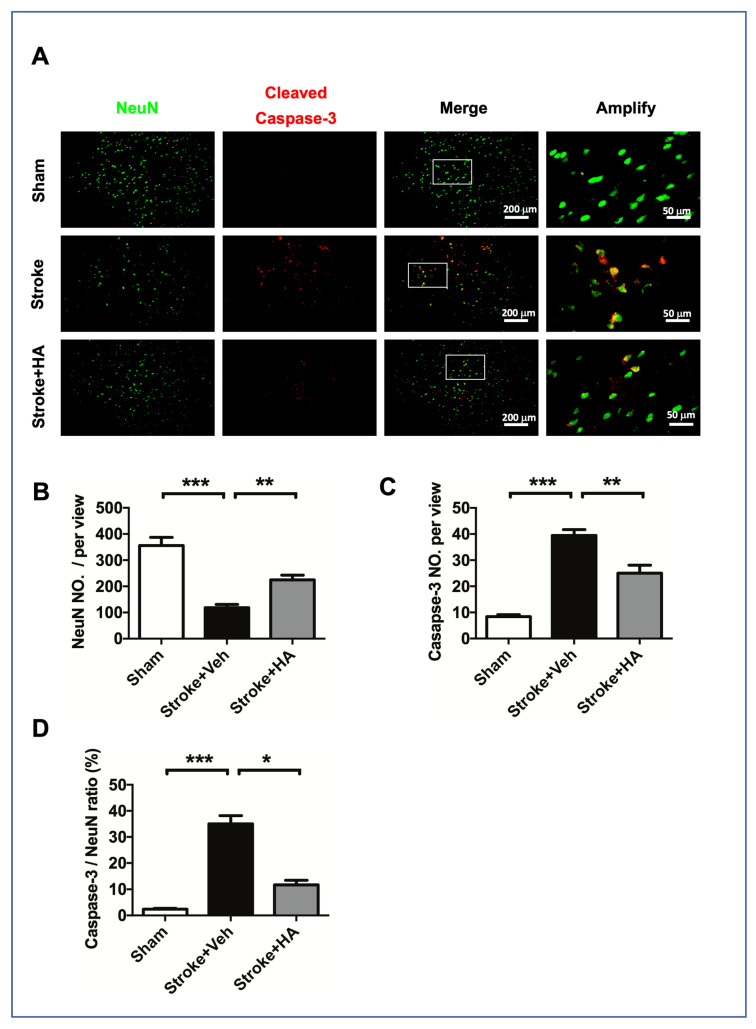
HA treatment alleviated neuronal apoptosis in the brain of rats after ischemic stroke. (**A**) Representative figures of immunofluorescent staining for NeuN and cleaved caspase-3 were conducted in rats with brain ischemia 3 days after stroke. (**B**) Quantified data of mean cell number with NeuN immunoreactivity in each view. (**C**) Quantified data of mean cell number with cleaved caspase-3 immunoreactivity in each view. (**D**) Quantified data of mean cell number with double-positive immunoreactivity of NeuN and cleaved-caspase-3 in each view. Data quantification accumulated twenty image views across six brain sections per brain from cortex penumbra. *n* = 8. * Denotes *p* < 0.05, ** denotes *p* < 0.01, *** denotes *p* < 0.001.

To understand the role of HA in neuroprotection after ischemic insult, we conducted immunofluorescent staining for NeuN and cleaved caspase-3 3 days after the stroke to examine neuronal apoptosis. Our data showed that ischemic insult induced considerable brain damage, reduced the number of cells with NeuN immunoreactivity, and increased the number of cells with cleaved caspase-3 immunoreactivity (Figure 4A), but HA treatment could reverse this consequence. Furthermore, obtaining a count of immunoreactive cells revealed that HA treatment protected the surviving neuronal cells (Figure 4B) and prevented cell apoptosis (Figure 4C) in the ischemic brain. Importantly, obtaining a count of cleaved caspase-3 and NeuN double-positive immunoreactive cells revealed that HA treatment attenuates neuronal apoptosis after ischemic insult (Figure 4D). Consequently, our immunostaining results indicated that HA has a neuroprotective effect on the ischemic brain.

### 2.5. HA Attenuates DNA Damage after a Stroke

**Figure 5:**
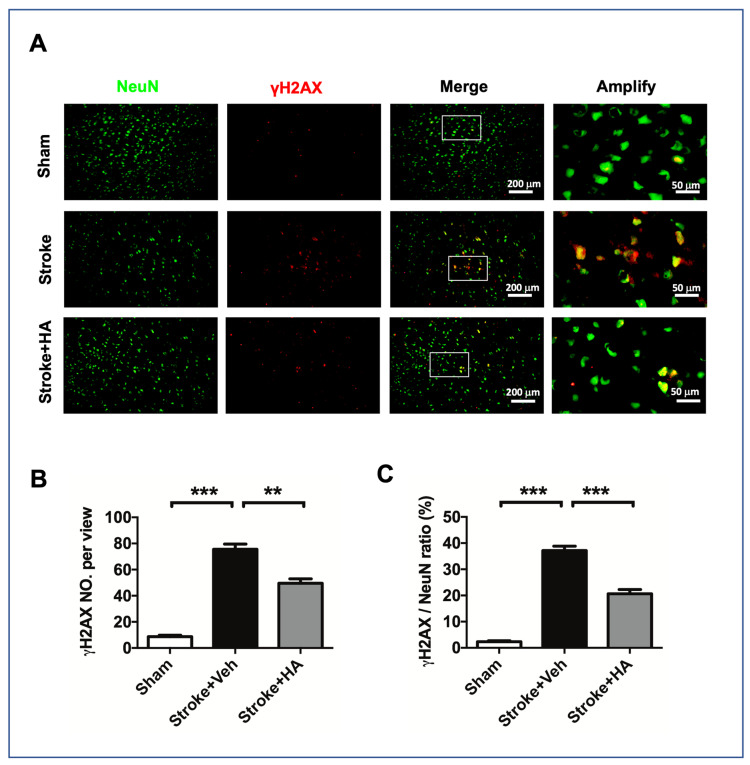
HA treatment attenuated neuronal DNA damage in the brain of rats after ischemic stroke. (**A**) Representative figures of immunofluorescent staining for NeuN and γH2AX were conducted in rats with brain ischemia 3 days after stroke. (**B**) Quantified data of mean cell number with γH2AX immunoreactivity in each view. (**C**) Quantified data of mean cell number with double-positive immunoreactivity of γH2AX and NeuN in each view. Data quantification accumulated twenty image views across six brain sections per brain from cortex penumbra. *n* = 8. ** Denotes *p* < 0.01, *** denotes *p* < 0.001.

To understand how HA has a neuroprotective effect on the ischemic brain, we further examined the DNA damage level in cerebral ischemia rats with immunofluorescent staining for NeuN and γH2AX to validate the underlying HDAC1-mediated mechanism in stroke pathogenesis. Our results showed that ischemic insult induced a considerable increase in the number of cells with γH2AX immunoreactivity compared with sham rats, but this consequence was not seen in HA-treated ischemia rats (Figure 5A). Furthermore, obtaining a count of γH2AX immunoreactive cells revealed that HA treatment significantly attenuates the increase in the number of cells with γH2AX immunoreactivity (Figure 5B) and prevents a positive ratio in neuronal γH2AX presentation (Figure 5C), suggesting that DNA damage attenuation is involved in HA-mediated neuroprotection.

### 2.6. HA Alleviates DNA Breaks after a Stroke

**Figure 6:**
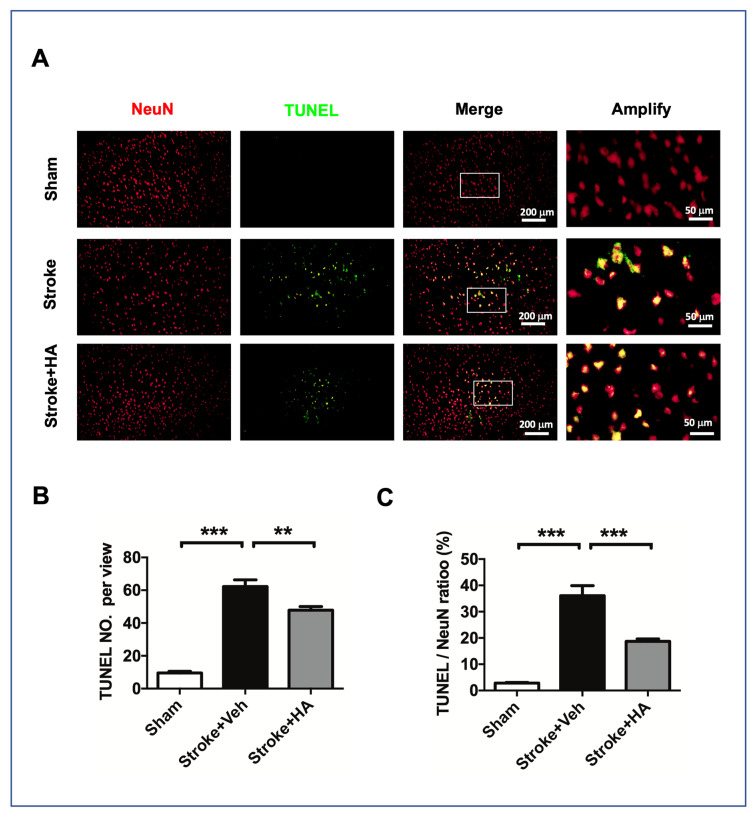
HA treatment ameliorated DNA breaks in the brain of rats after ischemic stroke. (**A**) Representative figures of TUNEL staining were conducted in rats with brain ischemia 3 days after stroke; immunofluorescent co-staining for NeuN was conducted to label neurons. (**B**) Quantified data of mean cell number with TUNEL signal in each view. (**C**) Quantified data of mean cell number with TUNEL signal and NeuN immunoreactivity in each view. Data quantification accumulated twenty image views across six brain sections per brain from cortex penumbra. *n* = 8. ** Denotes *p* < 0.01, *** denotes *p* < 0.001.

Given that HA treatment is involved in protection from DNA damage, we further investigated the effects of HA treatment on ischemic-insult-mediated DNA breaks by using terminal deoxynucleotidyl transferase dUTP nick-end labeling (TUNEL). To confirm the neuronal interplay in this assay, we conducted NeuN counterstaining to label neurons. The data showed that rats with ischemic insult exhibited increased levels of the TUNEL signal compared with sham rats, and HA treatment could attenuate this consequence (Figure 6A). In addition, our quantitative data indicated that treating cerebral ischemia rats with HA could reduce the total number of cells with a TUNEL-positive signal (Figure 6B) and attenuate neurons with a TUNEL-positive signal (Figure 6C), suggesting that HA treatment can protect neurons from DNA breaks after ischemic insult.

### 2.7. HA Promotes Recovery of Behavioral Deficits in Rats with Ischemic Stroke

**Figure 7:**
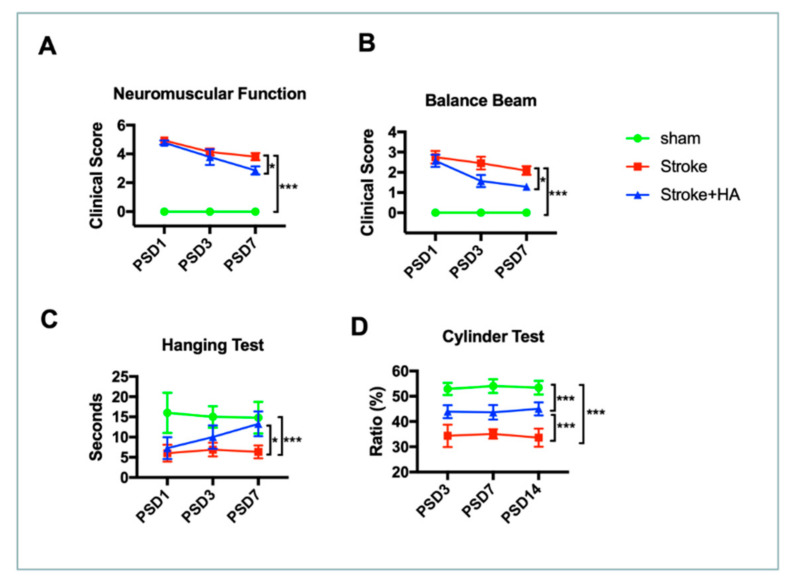
HA treatment improved the behavioral outcomes of rats after ischemic stroke. (**A**) Data of mNSS were acquired from rats with ischemic stroke at post-stroke day (PSD) 1, 3, 7. (**B**) Data of balance beam test were acquired from rats with ischemic stroke at PSD 1, 3, 7. (**C**) Data of hanging test were acquired from rats with ischemic stroke at PSD 1, 3, 7. (**D**) Data of cylinder test were acquired from rats with ischemic stroke at PSD 1, 3, 7. *n* = 8. * Denotes *p* < 0.05, *** denotes *p* < 0.001.

To determine the efficacy of HA in stroke therapy, we examined behavioral outcomes after stroke. First, we used a modified neurological severity score (mNSS) assessment to evaluate neurological and muscular functions at days 1, 3, and 7 after stroke. The data showed that an endothelin-1-induced ischemia model exhibited considerably damaged neurological function; in these models, mNSSs were lower than those in sham rats. However, HA treatment improved the mNSSs in rats with ischemic stroke (Figure 7A). In addition, HA further attenuated the motor deficits in balancing ability and forepaw strength; data from the balance beam test and the hanging test showed that cerebral ischemia rats with HA treatment progressively exhibited better performance over time (Figure 7B,C).

Moreover, to examine the effect of HA on the functional recovery of forepaw use at acute and subacute stages, we conducted cylinder tests on days 3, 7, and 14 after stroke. Our data showed that rats with cerebral ischemia could not sufficiently use their injured paw after ischemic insult. However, HA treatment improved the defects in injured paw use. A better ratio of using the diseased paw was observed, suggesting that HA treatment can promote functional recovery after ischemic insult. Together, these data confirmed the potential of using HA in the attenuation of behavioral outcome deficits.

### 2.8. HA Improves Recover of Synaptic Density in Rats with Ischemic Stroke

**Figure 8:**
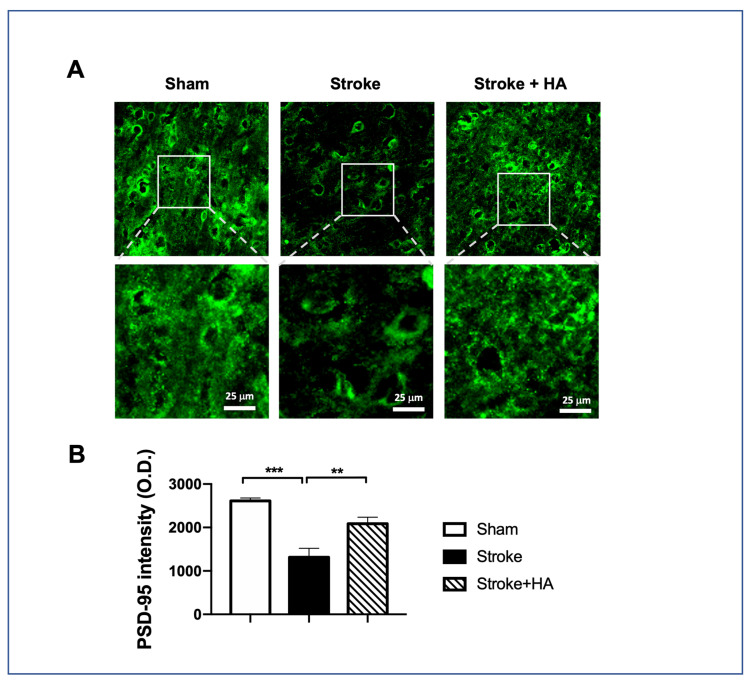
HA treatment improved synaptic density in the rats with cerebral ischemia. (**A**) Representative figures of immunofluorescent staining for PSD95. White boxes noted region with amplification at the lower panel. Data were acquired in the cortex penumbra. (**B**) Quantification of PSD95 immunoreactive density. *n* = 6 per group. ** Denotes *p* < 0.01, *** denotes *p* < 0.001.

Having confirmed the improvement of behavioral outcomes in cerebral ischemia rats by compound 5104434, we further examined synaptic plasticity after stroke using immunofluorescent staining for a post-synaptic marker, named PSD95, at PSD 7 (Figure 8A). Our data showed that rats with ischemic stroke exhibited significantly reduced synaptic density. In contrast, compound 5104434 treatment was better able to improve the neural plasticity in terms of post-synaptic density and an increased level of PSD95 in compound 5104434 treated rats with cerebral ischemia (Figure 8B). These data suggest that the recovered behavioral outcomes may result from an improvement of synaptic density.

## 3. Discussion

In this study, we confirmed that ischemic insult leads to the decreased enzymatic activity of HDAC1, and HA treatment with the compound 5104434 can specifically restore HDAC1 enzymatic activity and further protect the neuronal morphology from OGD damage. Furthermore, using a cerebral ischemia model of rats, we demonstrated that HA treatment with the compound 5104434 could provide a neuroprotective effect to the cerebral ischemic brain by ameliorating neuronal loss, apoptosis, DNA damage, and DNA breaks. Accordingly, the behavioral outcomes of neuromuscular response, balance, forepaw strength, and forepaw function recovery improved with HA treatment.

Ischemia-induced oxygen depletion can cause a spectrum of events in the pathogenesis, such as accumulation of oxidative stress, Ca^2+^ overload, and glutamate excitotoxicity [37,38,39]. These events may trigger the DNA breaks and damage, further promoting necrosis or apoptosis of injured neurons after ischemia [40,41]. Similarly, this study found an increase in γH2AX and TUNEL signals in our ischemia rat model, which considerably decreased after treatment with compound 5104434. Accordingly, we speculate that attenuation of DNA damage is the main mechanism of neuroprotection. In intracellular cascades following ischemic insult, γH2AX is phosphorylated with ataxia-telangiectasia mutated kinase to form phosphor-γH2AX during the initial cellular reaction to DNA double-strand breaks (DSBs) [42,43]. Additionally, γH2AX formation is dependent on chromatin modification and initiates the DNA repair response [44]. Accordingly, γH2AX behaves as a marker of DNA DSBs. In addition, TUNEL staining conducted herein detected the DNA breaks formed when DNA fragmentation occurred in the last phase of apoptosis. In this regard, HDAC1 is enrolled and distributes to DNA break sites for DNA repair [45,46]; HDAC1 also assists the deacetylation of DNA repair protein by interaction with sirtuin 1 and FUS [47,48], and it also modulates DNA repair by interactions with checkpoint kinases ATM/ATR [49,50,51] and DNA repair protein ATE [52]. These findings of HDAC1 involved in DNA repair may mechanistically support the efficacy of compound 5104434 in the potential treatment of strokes.

Each HDAC member modulates specific target genes, which can maintain or inactivate brain function [53]. However, the specific genes modulated by each HDAC member in the brain remain elusive. Generally, they play several crucial roles in ischemic damage and brain regeneration. For instance, HDAC1 acts as a molecular switch between neuron survival and death and a regulator in mood disorders. HDAC3 controls neuronal cell death through interaction with HDAC1. HDAC4 is associated with risks of autism spectrum disorder, neuronal cell survival, synaptic plasticity, and memory depression. HDAC5 controls the negative regulation of BDNF expression, which is upregulated in cocaine addiction. HDAC7 contributes to neuroprotection against apoptosis. HDAC6 is upregulated in a Rett syndrome model, has a negative effect on BDNF trafficking, and is involved in T-cell regulation in neuroinflammation after brain ischemia [54]. Remarkably, HDAC1 participates in modulating neurotoxic and neuroprotective responses by shuttling between cytosol and the nucleus and interacting with distinct target molecules. In the epigenetic modification under a diseased model of multiple sclerosis, serine phosphorylation of HDAC1 leads to its exporting to the cytosol. It induces neurotoxicity that disrupts axon transport and mitochondrial function in diseased mice [55,56]. Additionally, HDAC1 has been reported, which tat modulates neuroprotection by interaction with HDRP, sirtuin 1, and FUS under normal conditions [18,57,58]. In the present study, we further characterized the role of HDAC1 in stroke pathogenesis. It is involved in neuroprotection and DNA damage [21]. We found that while HDAC1 enzymatic activity was selectively recovered, the HDAC1-dysfunction-mediated damage was attenuated.

We found parallel studies that have reported that transient middle cerebral artery occlusion induced a progressive decrease in the cortical mRNA level of HDAC1 [59] and improved HDAC1 function protection against DNA damage in a forebrain ischemia model [17]. However, opposite findings were also reported, showing that OGD leads to a transient induction of HDAC1 mRNA and apoptosis in primary cortical neurons and that HDAC1 siRNA counteracted the cell death [60]. HDAC1 overexpression restored infarct volume through M1 microglial polarization induction in a transient MCA occlusion model [61]. We suspect that the inconsistent expression of HDAC1 after ischemic insult is a result of differences in animal models and observation time points used, and further evaluation and clinical evidence from a human sample are needed. In this study, we found that HDAC1 activity decreased in neurons exposed to OGD, and HA treatment with compound 5104434 can restore reduced HDAC1 enzymatic activity. Furthermore, in vivo assessments support its benefit in neuronal protection and behavioral outcomes. Thus, HDAC1 deregulation is crucial in stroke pathogenesis, and the compound 5104434 possesses therapeutic potential in attenuating brain damage and brain functionality loss.

Regarding pan-HDAC inhibitors, several kinds of nonselective inhibitors were reported with therapeutic potential in the brain, such as valproic acid, sodium butyrate, suberoylanilide hydroxamic acid, suberoylanilide hydroxamic acid, and trichostatin [62] Although treatment of HDAC inhibition potentially functions to recover perturbations in histone acetylation homeostasis and transcriptional activity to disease or disease-modifying genes, further functional evaluations are necessary to understand the mechanisms using selective approaches entirely. Interestingly, entinostat (MS-275) with selective inhibition to class 1 of HDAC members was identified, exhibiting beneficial effects in disease outcomes [63,64]. Furthermore, the interaction of HDAC1 and HDAC3 is involved in neuronal survival modulation66, and HDAC1 can further interact with histone deacetylase-related protein (HDRP), a truncated form of HDAC9, and contributes to neuroprotection [20]. HDAC1 can further interact with histone deacetylase-related protein (HDRP), a truncated form of HDAC9, and contributes to neuroprotection [20]. Comparing to the current study, we support the essential role of HDAC1 in the modulation of neuron viability, and we additionally provide a selective evaluation of HDAC1 in stroke pathogenesis. Thus, our data additionally extend the knowledge of HDAC1 underlying enzymatic activation.

Currently, the available stroke therapies are limited, particularly for efficient drug development. Here, we have provided evidence regarding the therapeutic potential of compound 5104434 in ameliorating ischemic damage. This compound was first suggested to potentially possess a selective character to activate HDAC1 activity in 2008, when Kim et al. published their findings regarding HDAC1 [17]; however, the detailed feasibility and efficacy were proven in 2020, when Wu et al. validated this essential function [34]. They evaluated the effectiveness of the compound 5104434 in HDAC1 activity in a frontotemporal lobar degeneration mouse model, HDAC2, 3, 8. They identified that HDAC1 activity is selectively altered by dose-dependent assessments and confirmed its positive role in learning/memory with interaction with the compound 5104434. Because pan-HDACi can simultaneously inactivate several HDAC types, various HDACs may have divergent effects. Some of them may elicit contrary functions for neurons; additionally, other cell types may be affected by pan-HDACi in the brain. These are obstacles and considerable concerns in the development of a new drug or therapeutic approach. Although pan-HDACis can ameliorate stroke and traumatic brain injury outcomes, successful treatments in classic animal models have rarely translated into clinical trials [65,66]. This study herein provides evidence that a specific HDAC1 activator that solves these challenges will potentially extend the therapeutic window and possibly lead to the clinical application of the HDAC-based therapeutic approach in treating brain diseases. Due to the specificity of HDAC1 activation and security of drug toxicity confirmed in the current study and our previous work [34], a sufficient drug for stroke therapy is still an unmet medical need. Our data herein support that compound 5104434 is worth conducting a clinical trial for further evaluation.

In conclusion, our study showed that HDAC1 deregulation is involved in stroke pathogenesis. Compound 5104434 administration attenuated ischemic-insult-induced neuronal morphology collapse, neuronal loss, apoptosis, DNA damage, and DNA breaks. Furthermore, the behavioral outcomes of neuromuscular response, balance, and forepaw strength and use were ameliorated. Collectively, our data support the efficacy of compound 5104434 in stroke therapy, and clinical trials must be conducted for further evaluation.

## 4. Materials and Methods

### 4.1. Animal Experiments and Drug Administration

All the animal studies were approved by the Institute of Animal Care and Use Committee of I-Shou University and E-Da Hospital (IACUC-EDAH-108017, 6 August 2019; IACUC-EDAH-108037, 21 February 2021; IACUC-ISU-108015, 8 October 2019). All the experiments followed the ARRIVE guidelines (https://arriveguidelines.org/ (8 October 2019)). Adult male Sprague Dawley rats were purchased from the National Laboratory Animal Center of Taiwan or Lasco biotechnology company (Taipei, Taiwan). A total of 54 rats were used in this study, including 6 pregnant female rats. The rats were used for all experiments and weighed 250–300 g. Rats were allocated randomly to the following experimental groups: sham and ischemia insult with vehicle treatment and ischemia insult with selective HDAC1 activator (compound 5104434). The procedure to practice animal surgery was followed and modified from our previous work [67]. To induce the ischemic stroke model, the vasodilator peptide endothelin-1 was intracranially injected [21]. Briefly, total 2 μL of 100 pM endothelin-1 (Sigma, E7764; St. Lois, MO, USA) was stereotactic injected into the brain (AP 0, ML + 2.5, DV − 2.3; AP + 2.3, ML + 2.5, DV − 2.3; AP + 0.7, ML + 3.8, DV − 7.0) [68]. For sham surgery, sham rats were stereotactically injected with HBSS only. Compound 5104434 (ChemBridge; San Diego, CA, USA) was dissolved in DMSO with a stock concentration of 3 mg/mL, and the administration (30 mg/kg/day in vivo) was conducted using daily i.p. injection [34]. In addition, the groups of sham control and stroke received PBS containing 1% DMSO as vehicle injection.

### 4.2. Primary Neuronal Culture, Oxygen Deprivation, and Neurite Outgrowth Assay

Primary neuron cultures were acquired from postnatal day 1 pup following a previous description [58]. Briefly, the pup brain was removed, minced, and enzymatically trypsinized by 0.25% trypsin (Thermo, Waltham, MA, USA, 25200056) in an incubator for 30 min at 37 °C. Next, the lysates were centrifuged and filtered through a 40 μm nylon mesh (Falcon 352340, Corning, NY, USA). We further seeded the single isolated cells on poly-D-lysine (Sigma P6407, Fremont, CA, USA)-coated dishes (2 × 10^5^ cells per mL) and cultured the neurons in a basal neural medium (Thermo 21103-049) containing 2% of B27 neural supplements (Thermo 17504044). We conducted OGD using a specialized chamber (CAT27310, Vancouver, BC, Canada). On day 10 of primary neuronal culture, we replaced the medium with serum-free OGD medium (Thermo 11430-030), and we subjected the neurons to a hypoxia chamber filled with oxygen-deficient air (90% N_2_, 5% O_2_, and 5% CO_2_) for 90 min at 37 °C in the incubator. The compound 5104434 was treated to culture neurons after OGD for 24 h. In quantifying the neurite outgrowth, MAP2 and DAPI double-positive cells were acquired by a high-throughput image screening system (Molecular Devices, Sunnyvale, CA, USA). We used the Neurite Outgrowth Module of the MetaXpress software (Molecular Devices) to analyze the number of neurons, length of total outgrowth per cell, and the number of processes and branches per cell.

### 4.3. Behavioral Tests

In the assessment of modified neurological severity score (mNSS), a focal scoring system for neurological severity score (NSS) was referenced and used to evaluate neurological outcomes of experimental rats on PSDs 1, 3, and 7 [69]. In the evaluations, the experiments were designed as follows: “Three grade scores were designed for each animal, with functional measures including gait, body symmetry, climbing, turning behavior, forelimb extension, compulsory circling, and sensory response”. Each evaluation was scored, and total scores were summarized as the performance assessment in the experimental time course. In the assessment of balance beam, balance ability assessment was referenced to the previous study and calculated the score depending on the performance [69]. We evaluated vestibulomotor function to examine the rat’s ability to balance on a narrow (30 × 1.5 cm) beam. In the hanging test, two forelimb wire hanging tests were referenced to the previous study and calculated the seconds of rat’s ability to stay on the grid [70]. We adopted a single 1 mm in diameter wire mounted between two ring stands. We prepared a standard mouse cage with cotton padding and placed it under the wire between the ring stands to protect the rat from damage. The time hanging on the wire was recorded, the average score from 3 trials per time was conducted and calculated. The cylinder test was conducted to evaluate forelimb deficits, followed by the previous paper [71]. The animal was placed in a transparent cylinder and assessed. When assessing behavior in the cylinder, the number of independent wall placements observed for the right forelimb and left forelimb was recorded. The ratio of using ill-site forelimb was quantified as R/(L + R)*100%. All rats underwent the pre-experiment of the behavioral test for two days before the surgery. The purpose was to acclimate the rats to the experimental environment and train the rats to adapt to the behavioral test situation to eliminate the rats that are not suitable for the experiment and reduce the practical interference.

### 4.4. ROS Assay, LDH Assay, HDAC1 Activity Assay

Brain extracts were prepared from the brain tissue to analyze protein expression levels following ischemia insult and drug treatment (bregma: +3 to −1 mm). We adopted a brain slicer to slice the brain to prepare the sample. The detailed protocol for Western blotting followed a previous study [72]. We purchased ROS and LDH assay kits from BioVision K936-100-250 and K726 (Milpitas, CA, USA) and followed the manual’s instructions. We bought an activity assay kit from BioVision (K342-25) for HDAC1 enzymatic activity evaluation. We isolated the brain and prepared the brain lysates for nuclear protein extraction (Millipore, Burlington*,* MA, USA; Catalog No. 2900). Then, we conducted HDAC1 immunoprecipitation overnight at 4 °C for the isolate, specifically HDAC, and performed enzymatic activity assay.

### 4.5. Infarct Volume Assessment

We adopted 2,3,5-triphenyl tetrazolium chloride (TTC; Sigma, T8877) to quantify the ischemia infarct volume. The procedures were conducted as we previously reported [73]. Briefly, we euthanized the rats, removed the brains, and separated the brain into 72 mm-thick slices using a rat brain slicer (World Precision Instruments, Sarasota, FL, USA). Next, we performed TTC staining to the brain slices using 0.2% TTC at 37 °C, and we fixed the TTC stained brain slices using a 4% paraformaldehyde solution. In the data quantification, we adopted ImageJ (ver2.1, NIH) to calculate the infarct area. Data for total infarct volume were accumulated from 6 TTC-stained brain sections in each rat.

### 4.6. Immunofluorescent Staining

We conducted IF staining followed our previous working procedure [74]. The rats were anesthetized and perfused with PBS and 4% paraformaldehyde to prepare the tissue for IF staining. Then, the brain was immersed in a 4% PFA solution for two h and dehydrated by gradient concentrations of sucrose. We collected the brain slices from bregma +2 to −4 mm and sliced the cryo-tissues at ten μm per section; one of three sections was composed and adhered to the slide. Thus, we accumulated ten slides from a single rat brain, six sections attached to a slide. We quantified twenty image views at least across six sections from cortex penumbra. After preparing cryosections, the brain sections were hybridized with a series of primary antibodies, including NeuN (Millipore, Catalog# MAB377) and γ-H2AX (Millipore, Catalog# 05636). To perform the chromogenic reaction, we adopted the secondary antibodies, AlexaFluor-conjugated secondary antibodies (Thermo, Waltham, MA, USA). Then, the brain sections were stained with DAPI and mounted under coverslips by using a mounting medium (Dako, Glostrup, Denmark). We quantified the immunoreactive cells and positive signals using ImageJ by setting a threshold for the intensity of immunoreactivity [72].

### 4.7. Neuron Primary Culture, Neurite Outgrowth Assay

Primary neurons were cultured from postnatal day 1 rat pups following procedures previously described [72]. Briefly, the pup brain was removed after decapitation, was minced by scissors, and trypsinized by 0.25% trypsin (Thermo 25200056) incubation for 30 min at 37 °C. Next, the lysates were centrifuged and filtered through a 40 μm nylon mesh (Falcon 352340, Corning, NY, USA). The isolated cells were seeded onto poly-D-lysine (Sigma P6407, Fremont, CA, USA)-coated dishes and cultured in a basal neural medium (Thermo 21103-049) containing 2% B27 neural supplements (Thermo 17504044). The cultured neurons performed neuritis outgrowth along the developmental days and were subjected to oxygen and glucose deprivation (OGD) at day 9 while they displayed matured pattern of neuritis complexity. We screened MAP2 and NeuN double-positive cells by the ImageXpress System (Molecular Devices, Sunnyvale, CA, USA) to quantify the neurite outgrowth. The Neurite Outgrowth Module of the MetaXpress software (Molecular Devices) was used to analyze the number of neurons, length of total outgrowth per cell, and the number of processes and branches per cell.

### 4.8. Statistics

All data are presented as the mean ± SEM. All data were normally distributed and were analyzed by one-way ANOVA with post hoc Tukey’s test for multiple-group comparison or by Student’s *t*-test for 2-group comparison. Differences with *p* < 0.05 were considered statistically significant.

## Data Availability

The data presented in this study are available on request from the corresponding author.
